# Hemithyroidectomy increases the risk of disease recurrence in patients with ipsilateral multifocal papillary thyroid carcinoma

**DOI:** 10.3892/ol.2013.1202

**Published:** 2013-02-19

**Authors:** XIAOLONG LI, CUI ZHAO, DANDAN HU, YANG YU, JIN GAO, WENCHUAN ZHAO, MING GAO

**Affiliations:** 1 Department of Head and Neck Tumor, Tianjin Medical University Cancer Institute and Hospital, Tianjin 300060, P.R. China;; 2 School of Medicine and Dentistry, James Cook University, Douglas, Queensland 4878, Australia

**Keywords:** papillary thyroid cancer, multifocal, recurrence, hemithyroidectomy

## Abstract

Papillary thyroid carcinoma (PTC) is often clinically multifocal. In this study, the clinicopathological characteristics of a total of 347 PTC patients treated between 2006 and 2007 were investigated in order to assess the risk factors for tumor recurrence in patients with multifocal PTC. Of all the PTC cases reviewed, 35 (10%) were categorized as multifocal PTC. Patients with multifocal PTC were significantly more likely to have extrathyroidal extension, lymph node metastases and disease recurrence (P<0.05). Hemithyroidectomy resulted in a significantly higher incidence of tumor recurrence in patients with ipsilateral multifocal PTC compared with unifocal PTC patients (P<0.01). In conclusion, hemithyroidectomy was associated with tumor recurrence in patients with ipsilateral multifocal PTC but not those with unifocal PTC. Hemithyroidectomy should only be carried out after careful deliberation when involving patients with ipsilateral multifocal PTC.

## Introduction

Papillary and follicular carcinoma are defined as differentiated thyroid carcinomas derived from thyroid follicular epithelial cells and account for >90% of all thyroid cancers (1–3). Papillary thyroid carcinoma (PTC) is significantly more common than follicular thyroid carcinoma and is characterized by a high rate of lymph node metastases (4–9). The incidence of multifocal PTC has been increasing rapidly (10,11).

There has been significant debate as to whether multifocal PTC patients should undergo aggressive surgical therapy. Certain studies have reported that PTC tumors should receive bilateral thyroidectomy and radioablation of the remaining tissue according to the finding that papillary cancer foci may have independent origins (10,12,13). Others have suggested that this treatment is too aggressive since multicentricity is not considered to be a prognostic determinant for the categorization of tumors as low or high risk (14). Furthermore, there is no consistent opinion on the treatment of central neck compartment lymph node metastases. Although the significance of therapeutic central neck dissection (CND) is undisputed, there is no consensus on the role of elective CND in clinically node-negative patients with multifocal PTC (15). To investigate this issue, a retrospective study of 347 cases of PTC, including 35 of multifocal PTC, was performed to determine the risk factors for multifocal PTC relative to known prognostic variables.

## Materials and methods

### Patients

A single institution retrospective review of patients with PTC was performed. The clinical data of a total of 347 patients treated for PTC between January 2006 and December 2007 at Tianjin Medical University Cancer Institute and Hospital (Tianjin, China) were investigated. None of the patients had a history of prior head and neck irradiation. All the patients underwent planned total thyroidectomy or hemithryoidectomy and CND or lateral neck dissection. Data on gender, age, histological variants, extrathyroidal extension, lymph node metastases and recurrence were available for all 347 patients. The present study was approved by the Tianjin Medical University Cancer Institute and Hospital Institutional Review Board. Written informed consent was obtained from the patients.

### Treatment and follow-up

Prior to surgery, every patient underwent a CT scan of the head and neck and an ultrasonographic evaluation of the thyroid and cervical lymph node. Fine-needle aspiration (FNA) of the primary tumor or intraoperative examination of frozen tissue sections was performed routinely to obtain the diagnosis of the primary disease. Frozen sections (2–3) were regularly analyzed depending on the tumor size. The surgical procedure was determined according to the results of the preoperative and intraoperative evaluations. Total thyroidectomy was performed on patients with any of the following conditions: i) tumor size >1 cm; ii) extrathyroidal extension; iii) palpable lymph nodes; and iv) bilateral carcinomas or ipsilateral disease with nodules in the contralateral lobe. Hemithyroidectomy was only performed on patients with ipsilateral disease and none of the four risk conditions. Central neck dissection (CND) was routinely performed on every patient. Lateral neck dissection was performed when the lateral nodes were positive.

All thyroid carcinomas were pathologically classified according to World Health Organization (WHO) criteria. The mean follow-up period was 4.4±0.2 years at the end of 2011. Ultrasonographic evaluation was performed on all patients in the first month after surgery. Every six months, ultrasonography was used for disease recurrence detection. Thyroglobulin (Tg), TSH and anti-Tg antibody measurements were also performed on patients who underwent total thyroidectomy. Recurrence was diagnosed by cytological or pathological examinations after follow-up surgery. Total thyroidectomy was performed in patients with recurrence, followed by radioactive iodide (^131^I) treatment. Lateral neck dissection was performed in patients with lymph node recurrence. Recurrences were identified in six patients who underwent hemithyroidectomy. All the recurrences were detected in newly developed nodules in the remaining thyroid tissue. Lateral lymph node recurrence was detected in two patients who underwent total thyroidectomy.

### Statistical analysis

Statistical analysis was performed using SPSS software (version 13.0; SPSS Inc., Chicago, IL, USA). Chi-squared analysis was used to compare frequencies between groups. The Kaplan-Meier method was used to analyze recurrence in a time-dependent manner. P<0.05 was considered to indicate statistically significant differences.

## Results

### Patient characteristics

In the present retrospective study, 347 patients (106 males/241 females) with PTC were included. The mean age at diagnosis was 46.6±2.1 years (range, 16–74 years). Multifocal PTC occurred in 10% of patients (n=35) and 90% (n=312) had unifocal PTC. Hemithyroidectomy was performed in 41% of unifocal PTC patients (n=128) who were considered to be low-risk patients at diagnosis. Of the patients with ipsilateral multifocal PTC who were considered to be low-risk patients, 20% (n=7) underwent hemithyroidectomy. Total thyroidectomy was performed for the remaining 59% of unifocal PTC and 80% of multifocal PTC patients. Lateral neck dissection was performed in 7% of patients (n=24) with positive lateral lymph nodes.

### Clinicopathological parameters of unifocal and multifocal PTC

Table I shows the clinicopathological parameters of unifocal and multifocal PTC. Age, gender, T stage, histological variants, extrathyroidal extension and lymph node metastases were comparable between the two groups. There was no significant difference between unifocal and multifocal PTC with regard to age, gender, Hashimoto thyroiditis and the tall cell variant, an aggressive PTC variant. However, patients with multifocal PTC were significantly more likely to have extrathyroidal extension. Of the patients with multifocal PTC, 65.7% exhibited extrathyroidal extension compared with 47.8% of patients with unifocal disease (P=0.04). Furthermore, a significant difference was identified in the rate of lymph node metastases between the two groups, with metastases occurring in 77.1% of multifocal PTC patients vs. 56.4% of unifocal PTC patients (P=0.02). Higher T stages at diagnosis were also observed in patients with multifocal PTC. Of the multifocal PTC patients, 71% were diagnosed with stage T3/4, which was significantly higher compared with the unifocal PTC patients (52%; P=0.03). Moreover, the tumor recurrence rate of multifocal PTC was extremely high compared with unifocal PTC. Recurrences were identified in 14% of multifocal PTC patients compared with <1% of patents with unifocal PTC (P=0.00).

### Recurrence in multifocal PTC

To identify which factors account for recurrence in multifocal PTC, the clinicopathological factors affecting tumor recurrence in patients with multifocal PTC were studied and are shown in Table II. There was no significant difference between patients with and without tumor recurrence with regard to age (P=0.13), gender (P=0.65), Hashimoto thyroiditis (P=0.32), lymph node metastases (P=0.32) and the tall cell variant (P=0.55). Notably, recurrence was most likely to occur in patients without extrathyroidal extension and with stage T1/2 tumors. Recurrences were diagnosed in 33.3% of patients without extrathyroidal extension and 40% of T1/2 patients within the 5-year follow-up period (P=0.02, P=0.01, respectively). These patients were considered to be low-risk. Hemithyroidectomy was performed if the patients had ipsilateral disease at diagnosis and none of the following conditions: i) extrathyroidal extension; ii) palpable lymph nodes; and iii) bilateral carcinomas or ipsilateral disease with nodules on the contralateral lobe. Surgical treatment was supposed to be involved with disease recurrence in this regard. In the present study, there were seven ipsilateral multifocal PTC patients who were defined as low risk and underwent hemithyroidectomy. Recurrences were identified in 57% of patients undergoing hemithyroidectomy compared with 3.6% of patents who underwent total thyroidectomy (P=0.00). This showed that hemithyroidectomy resulted in more frequent recurrence in multifocal PTC patients. This result was consistent with the observation that recurrences were most likely to be identified in low-risk multifocal PTC patients, since hemithyroidectomy was only performed in the low-risk group.

### Involvement of hemithyroidectomy in recurrence among unifocal PTC patients

To identify whether hemithyroidectomy also resulted in more frequent cases of recurrence in unifocal PTC patients, the number of patients with recurrent PTC was obtained and the patients were grouped by hemithyroidectomy and total thyroidectomy (Table III). All recurrences were located in the remaining thyroid tissue in the patients who underwent hemithyroidectomy. Recurrence of the lateral lymph nodes was detected in 2 patients who underwent total thyroidectomy. The recurrence rate of the multifocal PTC patients who underwent hemithyroidectomy was significantly higher than that of the unifocal PTC patients (57.1 vs. 1.6%, P=0.00). However, there was no significant difference between the multifocal and unifocal PTC groups who underwent total thyroidectomy (3.6 vs. 0.5%, P=0.12).

### Comparison of multifocal PTC patients undergoing hemithyroidectomy and total thyroidectomy

To further understand the difference in the recurrence rate between multifocal PTC patients undergoing hemithyroidectomy and patients undergoing total thyroidectomy, recurrence was analyzed in a time-dependant manner. Recurrences were identified in four out of seven multifocal PTC patients who underwent hemithyroidectomy. The median recurrence time was 40.0±6.55 months. Only one case of recurrence was identified in the patients who underwent total thyroidectomy. The recurrence rate of the multifocal PTC patients who underwent hemithyroidectomy was significantly higher than that of the patients who underwent total thyroidectomy (P=0.00; Table IV).

## Discussion

PTC is characterized by increased multifocal disease, frequent clinical nodal metastases (20–50%) and an even higher rate of subclinical micrometastases (50–90%) (4–9). PTC is often clinically multifocal carcinoma that presents within a single thyroid gland or pluriform. The frequency of multifocal PTC is 30–40% (1–3). The frequency of multifocality in the present study was 10%, which is relatively low. The pathogenesis of multifocality remains unknown. Several factors, such as iodine intake and single nucleotide polymorphisms (SNPs), may be involved in the multicentricity of PTC. However, further research into the origin of multicentricity is required.

There are two unresolved issues in the treatment of multifocal PTC: the necessity of elective CND as opposed to therapeutic CND (15) and the necessity of total thyroidectomy as opposed to hemithyroidectomy (10,16), particularly in developing countries. The debate about how to treat clinically-negative lymph nodes has arisen as lymph node metastases are not considered to be prognostic factors for poor survival in well-differentiated PTC patients (17). However, certain studies have suggested that a high proportion (80–90%) of patients exhibit subclinical lymph node metastases (micrometastases) at the time of surgical intervention (6–9). Furthermore, multifocal thyroid disease has been associated with distant metastases in certain studies (18,19), suggesting that multifocal PTC carries an increased risk of metastases.

In the present study, the rate of lymph nodes metastasis was compared between unifocal and multifocal PTC. A significantly higher proportion of patients with lymph node metastases were identified in the multifocal PTC group, indicating that the risk of metastasis is correlated with the multifocality of the primary tumor. Since regional metastases in patients older than 45 years do indicate significantly negative prognoses (20), elective CND is likely to be most beneficial for multifocal PTC patients, particularly those classified as high risk, since follow-up surgery is considered to be difficult in the central compartment in cases of disease recurrence (21,22).

The necessity of total thyroidectomy as opposed to hemithyroidectomy has been another widely debated issue for several decades (23). Although the American Thyroid Association (ATA) has suggested that all patients with multifocal PTC should undergo total thyroidectomy, hemithyroidectomy is often performed on patients with multifocal PTC to reduce complications. This argument is based on the problem that these carcinomas are either intrathyroidal metastases from the primary tumor spreading through the lymphatic system or independent carcinomas. Activating mutations in BRAF are particularly common in adults with PTC. In a number of studies, the presence of BRAF mutations was associated with more aggressive clinical courses (24–26). Thus, analysis of the distribution of BRAF aids in understanding the origins of multifocal carcinomas.

A number of studies have suggested that multifocal carcinomas have independent origins due to the heterogeneous distribution of BRAF mutations in multifocal PTC (10–13). These findings indicate that any thyroid tissue remaining in patients with multifocal PTC after surgery may contain, or be likely to develop, additional cancer foci which may become recurrences (10). This is consistent with the present observation that the recurrence rate of the multifocal PTC patients who underwent hemithyroidectomy was significantly higher than that of the unifocal PTC patients (57.1 vs. 1.6%, P=0.00). Notably, according to the present data it was the low-risk multifocal PTC patients, not the high-risk patients, who had a higher recurrence rate. These patients were expected to be less vulnerable to disease recurrence and poor prognosis. The data in Tables III and IV suggest that this is due to the fact that only the low-risk patients in this study underwent hemithyroidectomy instead of total thyroidectomy. It also appears that the high-risk patients were able to avoid disease recurrence as total thyroidectomy was performed in this group of patients. The observation that only 1.6% of patients with unifocal PTC who underwent hemithyroidectomy developed recurrence supports the theory that multifocal carcinomas have independent origins. The development of new cancer foci in the remaining thyroid tissue leads to disease recurrence in multifocal PTC patients undergoing hemithyroidectomy.

Thus, the decision on whether to perform hemithyroidectomy for patients with ipsilateral multifocal PTC should only be made after careful deliberation. However, it is clear that certain papillary cancer foci may have the same origin since there are mutlifocal carcinomas with homogeneous distributions of BRAF mutations or lacking BRAF mutations (27). A randomized clinical trial may resolve this issue with more certainty.

## Figures and Tables

**Table I:** Clinicopathological factors of unifocal and multifocal PTC.

Clinicopathological factor	Unifocal PTC (n=312)	Multifocal PTC (n=35)	P-value
Age, years	48.3±2.4	45.1±2.7	0.86
Male:female ratio	96:216	10:25	0.79
Hashimoto thyroiditis, no. (%)	21 (6.7%)	3 (8.6%)	0.68
Extrathyroidal extension, no. (%)	149 (47.8%)	23 (65.7%)	0.04
Node metastases, no. (%)	176 (56.4%)	27 (77.1%)	0.02
Tall cell variant, no. (%)	25 (8.0%)	2 (5.7%)	0.63
T1/2:T3/4 ratio	149:163	10:25	0.03
Recurrence, no. (%)	3 (0.9%)	5 (14.3%)	0.00

PTC, papillary thyroid carcinoma.

**Table II:** Effect of clinicopathological factors on recurrence of multifocal PTC patients.

Clinicopathological factors	No. patients	No. recurrence (%)	P-value
Age, years			
<45	17	4 (23.5)	
≥45	18	1 (5.6)	0.13
Gender			
Male	10	1 (10.0)	
Female	25	4 (16.0)	0.65
Hashimoto thyroiditis			
Yes	3	1 (33.3)	
No	32	4 (12.5)	0.32
Extrathyroidal extension			
Yes	23	1 (4.4)	
No	12	4 (33.3)	0.02
Node metastases			
Yes	27	3 (11.1)	
No	8	2 (25.0)	0.32
Tall cell variant			
Yes	2	0	
No	33	5 (15.2)	0.55
T stage			
T1/2	10	4 (40.0)	
T3/4	25	1 (4.0)	0.01
Surgical treatment			
Hemithyroidectomy	7	4 (57.1)	
Total thyroidectomy	28	1 (3.6)	0.00

PTC, papillary thyroid carcinoma.

**Table III:** Effect of surgical procedures on recurrence of unifocal and multifocal PTC patients.

	No. unifocal PTC (%)	No. multifocal PTC (%)	P-value
Recurrence after hemithyroidectomy			
Yes	2 (1.6)	4 (57.1)	
No	126 (98.4)	3 (42.9)	0.00
Recurrence after total thyroidectomy			
Yes	1 (0.5)	1 (3.6)	
No	183 (99.5)	27 (96.4)	0.12

PTC, papillary thyroid carcinoma.

**Table IV:** Analysis of recurrence in a time-dependant manner according to hemithyroidectomy and total thyroidectomy.

	No. patients	No. recurrences	Median recurrence time (months)	P-value
Hemithyroidectomy	7	4	40.0±6.55	0.00
Total thyroidectomy	28	1	-	
